# Developmental programming of offspring adipose tissue biology and obesity risk

**DOI:** 10.1038/s41366-021-00790-w

**Published:** 2021-03-23

**Authors:** Amanda Rodgers, Amanda N. Sferruzzi-Perri

**Affiliations:** Centre for Trophoblast Research, Department of Physiology, Development and Neuroscience, Downing Street, University of Cambridge, Cambridge, UK

**Keywords:** Obesity, Fat metabolism

## Abstract

Obesity is reaching epidemic proportions and imposes major negative health crises and an economic burden in both high and low income countries. The multifaceted nature of obesity represents a major health challenge, with obesity affecting a variety of different organs and increases the risk of many other noncommunicable diseases, such as type 2 diabetes, fatty liver disease, dementia, cardiovascular diseases, and even cancer. The defining organ of obesity is the adipose tissue, highlighting the need to more comprehensively understand the development and biology of this tissue to understand the pathogenesis of obesity. Adipose tissue is a miscellaneous and highly plastic endocrine organ. It comes in many different sizes and shades and is distributed throughout many different locations in the body. Though its development begins prenatally, quite uniquely, it has the capacity for unlimited growth throughout adulthood. Adipose tissue is also a highly sexually dimorphic tissue, patterning men and women in different ways, which means the risks associated with obesity are also sexually dimorphic. Recent studies show that environmental factors during prenatal and early stages of postnatal development have the capacity to programme the structure and function of adipose tissue, with implications for the development of obesity. This review summarizes the evidence for a role for early environmental factors, such as maternal malnutrition, hypoxia, and exposure to excess hormones and endocrine disruptors during gestation in the programming of adipose tissue and obesity in the offspring. We will also discuss the complexity of studying adipose tissue biology and the importance of appreciating nuances in adipose tissue, such as sexual dimorphism and divergent responses to metabolic and endocrine stimuli. Given the rising levels of obesity worldwide, understanding how environmental conditions in early life affects adipose tissue phenotype and the subsequent development of obesity is of absolute importance.

## Introduction

Obesity is classed as a global epidemic by the World Health Organisation, with 13% of adults estimated to be obese in 2017 [1]. Although once only observed in high income countries, the rate of obesity is now increasing in prevalence in many middle and low income countries. The increased incidence of obesity represents a major health challenge as it increases the risk of developing other noncommunicable diseases, including type 2 diabetes [2], fatty liver disease, [3], cardiovascular diseases [4], and cancer [5]. In the UK, the cost to the national health service for obesity was £6.1 billion in 2015, and the wider economic impact was £27 billion [6]. Many factors contribute to the development of obesity. These include socio-economic factors and food marketing, which contribute to population risk, as well as genetic and lifestyle factors, which impact individual risk. However, environmental factors during early life development have the capacity to programme the structure and function of organs in the body, including the adipose tissue, the defining organ of obesity. Understanding how environmental conditions in early life impact adipose tissue and the subsequent development of obesity is of absolute importance.

The developmental origins of health and disease (DOHaD) encompasses a field of research that correlates long-term health and disease outcomes with early life conditions. This field was initiated by findings of an association between fetal malnutrition, and the development of metabolic diseases, like insulin resistance and obesity in adulthood [7, 8]. Extensions of this work using human population and experimental animal data have shown that exposure to a diverse array of suboptimal maternal environments, including nutritionally unbalanced diets, low oxygen availability, and high body mass index (BMI) during gestation, modifies the risk of the offspring developing obesity [9, 10]. The aim of this review is to present the findings of these studies. Particularly, the impact of a poor gestational environment on the development and function of the adipose and programming of obesity. It will first provide information on adipose tissue biology, including the differences and similarities between humans and the experimental animal species used in the DOHaD field, as well as describe sexual dimorphisms that exist in the adipose tissue within species. This background information is rarely considered and is of key importance when comparing findings to understand the developmental origins of obesity.

## Adipose tissues

Adipose tissue is distributed throughout the body. Although initially thought of as inert storage sites for triacylglycerol, adipose tissue is now recognized as a dynamic endocrine organ vital to whole body homeostasis and metabolism. In mammals, adipose tissue is categorized as white adipose tissue (WAT) and brown adipose tissue (BAT). While both types are specialized to store lipids, they essentially have antagonistic functions. WAT acts as an energy reservoir, whereas BAT acts as an energy combustion site. Their functions are carried out by mature white and brown adipocytes, respectively. In addition to adipocytes, adipose contains other cell types, like leukocytes, pericytes, and endothelial cells. Moreover, both adipose types are vascularised and innervated [11], connecting the tissue to whole body metabolic regulation.

BAT was initially thought to be unique to neonates, hibernators, and small mammals, which is unsurprising as its primary function is to produce heat and these animals are predisposed to temperature loss [12]. However, work in the last decades has found that BAT is present in all mammals, including adult humans [13, 14].

**Fig. 1:**
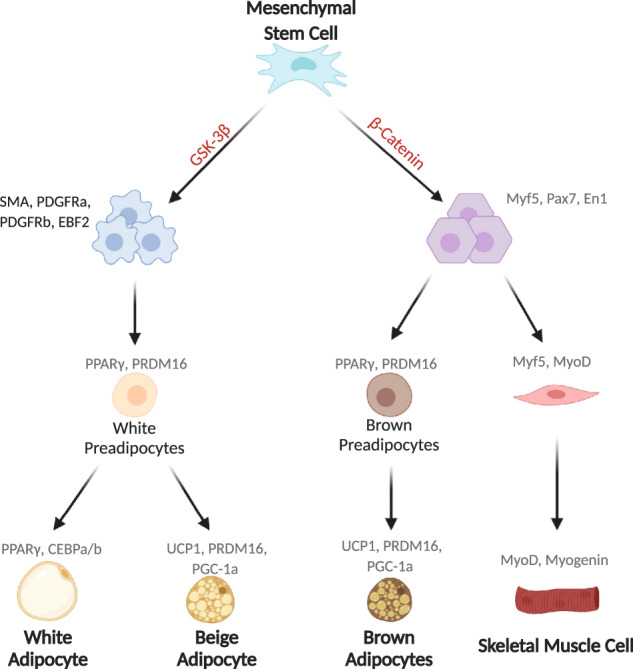
Molecular pathways controlling the differentiation of mesenchymal cells into brown and white adipocytes. Diagram depicts key stages in cell differentiation from mesenchymal stem cells to brown and white adipocytes, showing key cell markers at each cell stage. Brown and white adipocytes are derived from mesenchymal stem cells [156] and a number of factors control their differentiation. However, the GSK3β and β-catenin-WNT signaling pathways are particularly important for white adipocyte and brown adipocyte/myocyte lineage commitment, respectively [157]. A zinc finger transcriptional regulator, PRDM16 controls a bidirectional cell fate switch between skeletal myoblasts and brown adipocytes [158]. Differentiation of white adipocytes occurs down a separate lineage and adipogenesis of preadipocytes into mature adipocytes in WAT is controlled by transcriptional regulators, including peroxisome proliferator-activated receptor-γ (PPARγ) and CCAAT/enhancer-binding proteins (C/EBPs) [20–24]. C/EBPα and C/EBPβ are expressed early in the adipogenesis process along with certain zinc finger proteins, such as ZFP423, they are expressed shortly after commitment to the white adipocyte lineage and subsequently upregulate PPARγ [159]. These molecular factors then operate together to regulate the expression of other adipocyte-specific genes, resulting in the formation of mature adipocytes [159].

Brown adipocytes are derived from a common progenitor with myocytes (Fig. 1) and are more closely related to myocytes than to white adipocytes. Mature brown adipocytes store lipids in multiple small vacuoles and have an abundance of mitochondria, which are vital to heat production. Breakdown of fatty acids in brown adipocytes leads to an escalation of proton ions in the intermembrane space of mitochondria. UCP1, an uncoupling protein that is a key characteristic of brown adipocytes, uncouples this proton gradient from the ATP production by increasing the permeability of the inner mitochondrial membrane, leading to heat production [15]. In addition to brown adipocytes [16], BAT has many precursor cells, known as adipoblasts or preadipocytes, present in the tissue.

In contrast to brown adipocytes, white adipocytes have one large lipid droplet and few mitochondria. As the lipid droplet occupies the majority of the cell, white adipocytes have relatively little cytoplasm. White adipocytes are typically larger than brown (~100 µm in diameter versus 30 µm). White adipocytes differentiate from a separate lineage to brown adipocytes (Fig. 1 and [17]), although like BAT, WAT is full of progenitor cells. The presence of preadipocytes in WAT allows it to be highly plastic and capable of producing more mature white adipocytes when necessary [18]. For instance, when caloric intake is high, WAT increases its capacity for storing lipids, via both adipocyte hyperplasia and hypertrophy [19, 20]. WAT is also able to undergo a process known as “beiging”. Under certain conditions, such as cold exposure, some white preadipocytes can develop brown adipocyte characteristics, including multiple lipid droplets, increased mitochondrial density, and UCP1 expression [21]. These cells are known as brite or beige adipocytes and alter WAT physiology by affecting how it functions, and its hormonal output [22, 23].

### Adipose depots, development, and differences between species

**Fig. 2:**
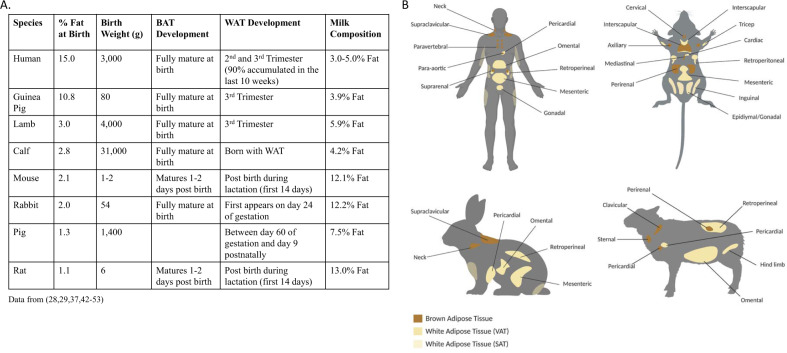
Adipose tissue in different model species. **A** Table comparing adipose development in different model species, showing average birth weight, average percentage fat at birth, timing of BAT and WAT development and average percentage fat in species milk composition. **B** Diagram depicting main adipose storage sites in different model species. *VAT* visceral adipose tissue, *SAT* subcutaneous adipose tissue.

The quantity of BAT and WAT at birth and in adulthood differs greatly between species (Fig. 2). Human infants are born exceptionally fat [24], with fat making up ~15% of a neonate’s body weight. The closest experimental species to humans in terms of fatness at birth is the guinea pig, with ~11% fat at birth [25], (Fig. 2A). There is also a wide variation in the fat content of milk between species, with most small species containing more fat compared to larger species, like humans (Fig. 2A and [24–38]).

Humans are born with WAT depots that develops during the final stages of gestation, with 90% being deposited during the last 10 weeks of gestation. Guinea pigs, rabbits, and sheep also accumulate WAT during the final third of gestation [39, 40]. During this time, there is an increased production of hormones, like insulin-like growth factors (IGFs) and leptin in the fetus that promote fetal adipose tissue growth [41–43]. However, mice and rats are born without WAT, which develops in the first 14 days post birth from progenitor cells in non-adipocyte structures [44]. Therefore, in terms of adipose development, the lactational period in rodents may be more equivalent to the third trimester of human pregnancy. Regardless, WAT depots tend to expand as age increases in the majority of species.

The function and location of WAT is similar across most species. Most WAT depots can be regarded primarily as nutrient reserves and small amounts of fat are distributed throughout the body in association with different organs. Ectopic fat stores can vary in their secretory output and function based on their associated organ. In the majority of mammals, the bulk of WAT is contained in the superficial fascia between and within muscles, below the peritoneum and in the bone marrow. WAT depots are often also subdivided into either subcutaneous adipose tissue, which lies under the skin, or visceral adipose tissue, which lies inside the abdominal cavity in association with internal organs.

BAT, however, is much more restricted in its distribution and in most species the largest quantity of BAT is concentrated around the neck and spine (Fig. 2B). In contrast to WAT, most animals are born with BAT and therefore develop it prenatally. There are also some differences in BAT differentiation across the main species studied. In rabbits, guinea pigs, lambs, and humans, BAT is fully mature and functional at birth, whereas in mice and rats, although BAT is present at birth, it only matures 1–2 days after birth. Moreover, in mice, rats, and guinea pigs, BAT depots, including the interscapular BAT remain throughout life with little reduction in size. Contrastingly, larger species, like rabbits, sheep, cows, and humans are born with BAT stores, but most of these are gradually replaced by WAT after birth [13, 45, 46].

### Sex differences in adipose tissue biology and obesity

Adipose tissue composition and location differs between sexes, with humans possibly displaying the most extreme example. The average human male body is comprised of 14% fat whereas the female body is comprised of 27% fat [47]. The disparity in adiposity can be observed as early as late gestation [48] and may indicate there are sex differences in mesenchymal cell fate decision towards the muscle versus adipocyte lineage.

There are also differences in localization of fat between sexes. Females typically have a gynoid fat distribution, where fat is stored around the hips and thighs, while males typically display an android form of fat distribution, where fat is stored around the waist [49, 50]. Increased adiposity in women produces a relatively benign form of obesity, whereas in males it is associated with higher risk for hypertension, insulin resistance, diabetes, dyslipidemia, and heart disease [51]. The higher risk associated with the android form of obesity is thought to be caused by leak of fatty acids from adipose into the portal vein, leading to accumulation of fat in ectopic organs and subsequent lipotoxicity. The differences in adipose location between sexes are thought to be largely caused by estrogen, as postmenopausal women develop a more android distribution.

Sexual dimorphism is far less documented in other species. Like humans, female rodents harbor greater fat mass compared to males [52, 53]. In addition, ovariectomized female mice demonstrate a similar patterning of adipose tissue to males [52]. Furthermore, unlike their male counterparts, several strains of female mice are relatively resistant to obesity on a high-fat diet [54, 55], but this protection can be removed by ovariectomy [56, 57]. Thus, fat patterning in adult females is influenced by reproductive hormones. There are also differences in BAT composition between sexes; in rodents and humans, females have a higher proportion of BAT compared to males. Thus, adipose tissue is a sexually dimorphic tissue, which is influenced by sex hormones and modifies the risk of obesity.

### Maternal nutritional restriction and offspring adiposity

Maternal nutritional restriction was the first type of dietary manipulation to be studied in the context of DOHaD. The long-term consequences of the 1944 Dutch famine was the subject of the original epidemiological studies that led to the DOHaD hypothesis. This work found that exposure to famine particularly in the first trimester of pregnancy was associated with an increased risk of the offspring developing obesity in adulthood [58, 59].

**Table 1:** Maternal nutritional and caloric restriction and its effect on offspring adiposity.

Study	Species	Maternal diet	Diet duration	Offspring diet	Offspring sex	Offspring age	Adipose structure	Adipose molecular changes	Fetal/Birth weight changes
Ashwell et al. [62]	Guinea pig	Control (Ad lib) Vs 50% caloric restriction	GD 30 until birth	NA	Not specified	Newborn	↓WAT mass ↑BAT mass	NA	↓ Birth weight
Bispham et al. [60]	Sheep	Nutrient requirement Vs control (Ad lib throughout)	GD 80–140	NA	Not specified	GD 140			↓ Fetal weight
		60% Nutrient restriction Vs Control (Ad lib throughout)	GD 28–80				↑Adipose tissue	↓ PPAR mRNA	
		60% Nutrient restriction then to requirement Vs control (Ad lib throughout)	GD 28–140				↑↑Adipose tissue	↑ PPAR mRNA and UCP2 mRNA	
Budge et al. [65]	Sheep	50% Nutrient requirement Vs control (100% requirement)	GD 10–45	NA	Not specified	GD 145	↓ Perirenal adipose tissue	↓ UCP1 mRNA in perirenal adipose tissue	↔ Fetal weight
Budge et al. [65]	Sheep	70% Nutrient requirement Vs control (100% requirements)	–GD60–GD 8	NA	Not specified	GD143	↔Perirenal adipose weight	↔ UCP1 mRNA	↔ Fetal weight
		70% Nutrient requirement Vs control (100% requirements)	GD 8–Term				↔ Perirenal adipose weight	↔ UCP1 mRNA	↓ Fetal weight
		70% Nutrient requirement Vs control (100% requirements)	–GD60–Term				↔ Perirenal adipose weight	↑ UCP1 mRNA perirenal adipose tissue	↔ Fetal weight
Long et al. [61]	Cow	70% Nutrient recommendation Vs control (100% nutrient recommendation)	GD45–GD 185	Standard diet	Both	16 months	↑Adipocyte size in SAT, mesenteric and omental depots	↑ FATP1 in SAT	↔ Birth weight
Tchoukalova et al. [63]	Baboon	70% Nutrient requirement Vs Control (Ad libitum)	GD 30–Term	NA	Both	GD 165	↑Adipocyte hypertrophy (males)	↓TBX15 mRNA (female only) ↑ PPAR-γ and FABP4, UCP1, PGC-1α and COXIV in differentiated adipocytes in vitro (male only)	↓ Fetal weight (male only)

*Ad Lib* ad libitum feeding, *BAT* brown adipose tissue, *GD* gestational day, *NA* not applicable, *SAT* subcutaneous adipose tissue, *VAT* visceral adipose tissue, *Vs* versus, *WAT* white adipose tissue.

Maternal nutrient and caloric restriction in a number of animal models has also been found to programme the offspring for increased adiposity ([60, 61] and Table 1). Moreover, there is evidence that this increase in adiposity is related to an increase in brown and beige adipose tissue in particular species, namely guinea pigs [62], as well as adipocyte hypertrophy in other species, like cows and baboons [61, 63]. Enhanced adiposity is also accompanied by changes in PPAR and UCP1/2 abundance in the adipose of offspring from undernourished mothers, however the timing of the maternal nutrient restriction seems to determine the nature of the molecular changes and level of adiposity [60, 63–65]. There are also programmed changes in the expression of mitochondrial-related genes, like PGC-1α and lipid handling genes, like FATP1 in the offspring adipose [61, 63]. Maternal nutrient restriction is often accompanied by decreased fetal growth or birth weight of the offspring [60, 62, 63, 65]. Thus, the greater propensity to become obese may reflect metabolic adaptations to optimize growth when nutrient supply is limited in utero, but may be mal-adaptive and lead to excess storage of nutrients when nutrient supply is not limited postnatally.

**Table 2:** Maternal low protein diets and its effect on offspring adiposity.

Study	Species	Maternal diet	Diet duration	Offspring diet	Offspring sex	Offspring age	Adipose structure	Adipose molecular	Whole body changes
Kim et al. [78]	Mice	Low protein (10%) Vs normal protein (20%)	–GD14–Weaning	HFD (45% fat)	Male	22 weeks	↑ Epididymal fat ↓ Perineal fat	Altered gene profile with adipokine and inflammatory pathways affected	↓Birth weight ↑Insulin sensitivity
Guan et al. [75]	Rats	Low protein (8%) Vs normal protein (20%)	GD0 Weaning	Chow	Male	130 days old	↑ Visceral adiposity	Altered expression of 650 genes in visceral adipose tissue:↑Carbohydrate, lipid, and protein metabolism, adipocyte differentiation, angiogenesis, and extracellular matrix remodeling pathways	↓Birth weight
Zhang et al. [76]	Rats	Low protein (8%) Vs normal protein (20%)	GD0–Weaning	Normal protein	Males	130 days old	↑ Rate of preadipocyte Proliferation	NA	↑ Visceral adiposity
Berends et al. [80]	Rats	Low protein (10%) Vs normal protein (20%)	Confirmation of pregnancy–Weaning	Cross fostered to normal protein mothers then chow	Male	22 days and 3 months old	↑ Adipocyte size	↓ AKT-2, IRS-1, p110β	
Bellinger et al. [84]	Rats	Low protein (9%) Vs normal protein (18%)	GD0–GD7	Chow	Males and females	9 months and 18 months	↔	NA	↔
			GD8–GD 14				At 18 months ↑ Abdominal fat ↓ Subcutaneous fat	NA	Hypophagia (males only)
			GD15–GD22				↑ Central fat (females only)	NA	Hypophagia (females only)
			GD0–GD 22				At 9 months ↓ Gonadal fat (males only) At 18 Months ↑ Abdominal fat ↓ Subcutaneous fat ↑Central fat (females only)	NA	Hypophagia (females only)
Claycombe et al. [83]	Rats	Normal protein (20%)	–GD21–Weaning	Normal fat (10% fat) Vs high fat (45% fat)	Male	12 weeks	↔	↔	↔
		Low protein (8%)		Normal fat (10% fat) Vs high fat (45% fat)			↑ Small adipocytes	↑ Igf2	↓ Insulin sensitivity
Xie et al. [82]	Rats	Normal protein (20%)	GD2–Weaning	Normal energy diet (3.84 Kcal/g) Vs high energy diet (4.73 Kcal/g)	Not specified	12 weeks	↔	↑ IL-6	↔
		Low protein (8%)		Normal energy diet (3.84 Kcal/g)			↓ Fat mass	↑ IL-6	
				High energy diet (4.73 Kcal/g)			↓ Fat mass ↓ CD68 + CD206 + cells in adipose	↑ IL-6	
Dumortier et al. [79]	Rats	Normal protein (20%)	0–Weaning	Chow Vs HFD (42% fat)	Male	8 weeks and 40 weeks	↔	↔	↔
		Low protein (8%)		Chow				↑ UCP1 in BAT (at 8 weeks)	
				HFD (42% fat)			↑ Interscapular BAT (at 8 weeks)	↑ UCP1 expression in BAT	At 40 weeks: ↑ Insulin resistant hyperglycemic ↑ Weight
Pinheiro et al. [77]	Rats	Low protein (5%) Vs normal protein (19%)	Birth–Weaning	Chow diet from weaning for F1 and F2	F2 offspring both male and female	6 months of age	↔	NA	Hyperglycemia, hyperinsulinaemia,↑ Insulin resistance
		Low protein (5%) Vs normal protein (19%)	GD0–Birth				↑ Fat mass (both F1 and F2 males)	NA	Hyperglycemia, hyperinsulinaemia, ↑ Insulin resistance, ↑ Birth weight
		Low protein (5%) Vs normal protein (19%) for entirety	GD 0–Weaning				↑ Fat mass (F1 and F2 males)	NA	
Tarry-Adkins et al. [72]	Rats	Low protein (8%) Vs normal protein (20%)	Duration of F1 generation	Chow after weaning at 21 days	Female	F2 at 3 months and 6 months	↑ Para-ovarian fat pad weight ↑ Adipocyte size with age	↑ PKC-ζ protein expression ↑ IL-1β protein and mRNA ↓ pAKTser473 Protein expression	
Pan et al. [71]	Pig (Landrace × Yorkshire crossbred sows)	Low protein (8%) vs standard protein (15–18%)	GD7–Weaning	Na	Male	4 weeks		↑ PPAR-γ mRNA, glucocorticoid receptor, ATGL, and HSL mRNA ↑ ATG7 and LC3 protein and mRNA ↓ Acetyl-CoA carboxylase and fatty acid synthase mRNA and protein↓ Triglycerides	↓ Birth weight
DuPriest et al. [74]	Pig (Yucatan microswine sows)	Normal protein (14%)	GD 80–Week 2 of lactation	Ad Lib Vs calorie restricted (75% Kcal)	Male and female	12 weeks	↔ Adipocyte size or adiposity	↔	↔
		Low protein (1%)		Ad Lib			↔ Adipocyte size or adiposity	↓ Adiponectin mRNA	↑ Fat deposition rate
				Calorie restricted (75% Kcal)			↔ Adipocyte size or adiposity	↓↓ Adiponectin mRNA ↓↓ TNF-α mRNA	↔
Nielsen et al. [73]	Sheep	Normal protein	GD 105–Term	Conventional hay diet Vs high fat high carbohydrate diet	Male and female	6 months and 2 years	↔	NA	↔
		Low protein (50% of the protein amount to NP)		Conventional hay diet			↓ Deposition of subcutaneous fat	NA	↓ Birth weight ↑ Appetite for fat
				High fat high carbohydrate diet			↓ Deposition of subcutaneous fat ↑ Visceral fat (females only)	NA	↓ Birth weight ↑ Appetite for fat
Micke et al. [81]	Cow	High protein (220–50% of recommended daily intake)	GD0–GD180	Normal energy diet	Male and female	680 days of age		↑ IGF1 mRNA (in females) ↑ LEP mRNA (in males)	↑ Birth weight
		High protein then low protein	GD 0–GD 93 then GD 94–GD 180					↑ IGF1 mRNA (in females) ↑ LEP mRNA (in males) ↑ IGF2 mRNA	
		Low protein then hight protein	GD 0–GD 180 then GD 94–GD180						↑ Birth weight
		Low protein (60–75% of reccomended daily intake)	GD 0–GD 180					↑ IGF2 mRNA	

*Ad Lib* ad libitum feeding, *BAT* brown adipose tissue, *GD* gestational day, *HFD* high fat diet, *NA* not applicable, *SAT* subcutaneous adipose tissue, *VAT* visceral adipose tissue, *Vs* versus, *WAT* white adipose tissue.

Symonds et al. found the timing of a nutrient restriction in sheep plays a role in determining the offspring outcome [26]. They found that exposure to maternal nutrient restriction up to day 110 of sheep gestation (term = 145 days) promoted adipose tissue deposition in the fetal lamb at term, whereas continuing the nutrient restriction after this time decreased adipose tissue mass. Bisham et al. [66] found that restoring maternal diets to the same as controls after day 80 of gestation further stimulated fat deposition in the fetus. Increased adiposity despite exposure to maternal nutrient restriction was accompanied by an increased abundance of IGF receptors, suggesting enhanced adipose tissue sensitivity to the anabolic effects of IGFs [67]. However, further information on the cellular, metabolic, and molecular changes governing alterations in offspring adiposity with maternal nutrient restriction, especially with regards to timing of insult, require investigation.

### Maternal low protein diets and offspring adiposity

Studying the impact of gestational exposure to maternal low protein foods, may help to understand the recent rise in the rates of obesity and other metabolic diseases in many countries [68]. Observational studies in humans have shown that low protein consumption during pregnancy correlates with fetal growth restriction [69]. In addition, studies in areas, such as South India where many women are vegetarian, have shown a correlation between reduced milk protein intake and low birth weight [70].

In contrast to the human studies, there is a large volume of animal studies that have investigated the effects of a maternal low protein diet on offspring adipose tissue (Table 2). These involve examining the effects of a low protein diet for the entirety of pregnancy, at particular stages in gestation and continuing the low protein diet during lactation and across generations. Some studies also expose the offspring to different nutrient manipulations postnatally. Overall, the available data suggest that maternal low protein diets have a complex effect on adipose tissue biology, with studies finding divergent molecular changes [71, 72].

Across various species, maternal low protein diet appears to have a two stage impact on offspring adiposity (Table 2). Maternal low protein diets tend to reduce offspring birth weight and adiposity in early life [71, 73], and may even be protective against the development of obesity in juvenile offspring fed a high fat or obesogenic diet postnatally [74]. However, offspring from low protein fed mothers accumulate greater amounts of fat as they mature [75, 76] and often display insulin resistance and hyperglycemia in adulthood [77, 78]. Furthermore, as they get older, offspring from protein restricted mothers have a greater sensitivity to the obesogenic effect and metabolic dysfunction induced by chronic exposure to high-fat diet [79]. However, data from pigs has shown that calorically restricting offspring exposed to a maternal low protein diet during gestation prevents the programming of increased adiposity postnatally, although molecular changes are still evident in the adipose tissue [74].

Studies in rats have shown that the effect of a maternal low protein diet on offspring adiposity is still observed even when the offspring is cross-fostered [80]. Moreover, there are greater increases in fat mass in first and second generation males from mothers that had been subjected to protein restriction during just gestation rather than gestation and lactation [77]. These data highlight the importance of nutrient supply in utero in determining offspring adiposity, with possible consequences for the offspring in subsequent generations. Indeed in sheep, even maternal protein restriction during very late gestation can predispose offspring for enhanced visceral adiposity in postnatal life [73]. Work in cows also suggest that the timing of maternal protein intake imbalance is an important determinant of offspring adiposity [81].

Across the species surveyed, changes in offspring adiposity are accompanied by alterations in adipocyte size or number, insulin signaling and metabolic protein levels, including PPARγ, as well as inflammatory markers and leukocytes in the adipose tissue of the offspring born from low protein diet-fed mothers [71, 72, 78, 82]. Importantly there are changes in leptin, adiponectin, and other important hormones like IGFs in the adipose of offspring [81, 83], which may contribute to their enhanced adiposity postnatally. Finally, although less explored, offspring of low protein diet-fed mothers show increased BAT thermogenic activity and UCP1 expression [79], which may provide some explanation for their initial protection against high-fat-diet-induced obesity as young adults.

There is some evidence to suggest that adipose tissue biology and obesity in female and male offspring may be differentially programmed by exposure to a maternal low protein diet during development [73, 81, 84] however, further work is required.

### Maternal obesity/obesogenic diets and offspring adiposity

With the incidence of obesity among women of child-bearing age rising, assessing the effect of maternal obesogenic diets on offspring metabolic outcomes is particularly relevant. In humans, maternal obesity is associated with an increased risk of their child having increased adiposity during infancy and obesity in adulthood [85]. Compelling evidence showing a role for developmental programming rather than genetic factors in the maternal transmission of obesity comes from studies of women who have undergone bariatric surgery to reduce weight; siblings born of women post surgery displayed a reduced prevalence of obesity compared to those born before surgery [86, 87].

Animal studies assessing the effect of maternal obesity/obesogenic diets on offspring adiposity broadly seem to support human association data and show consistent outcomes across species (Table 3). Offspring from obese/high-fat diet-fed mothers show increased adiposity [88–90] and adipocyte hypertrophy postnatally [89, 91]. They also often show increased inflammatory marker expression and a higher infiltration of macrophages and other immune cells in their adipose tissue [89, 91, 92], which may reflect lipotoxicity and likely contributed to the glucose intolerance seen in such offspring. Indeed, offspring from mothers fed obesogenic diet often also demonstrate decreased insulin signaling protein levels in their WAT [89, 93].

**Table 3:** Maternal high fat and obesogenic diets and its effect on offspring adiposity.

Study	Species	Maternal diet	Diet duration	Offspring diet	Offspring sex	Offspring age	Adipose structure	Adipose molecular	Whole body changes
Summerfield et al. [89]	Mice	HFD (60% kcal from fat) Vs normal diet (10% kcal from fat)	–GD84–GD63	HFD for 12 weeks after weaning	Male	15 weeks	↔	↔	↔
		HFD (60% kcal from fat) Vs normal diet (10% kcal from fat)	–GD 84–GD35				↑ Adipocyte hypertrophy ↑ Macrophage infiltration	↑ Inflammatory cytokines	↓ Glucose tolerance
		HFD (60% kcal from fat) Vs normal diet (10% kcal from fat)	–GD84–GD7				↑ Adipocyte hypertrophy ↑ Macrophage infiltration	↑ Inflammatory cytokines	↓ Glucose tolerance
		HFD (60% kcal from fat) Vs normal diet (10% kcal from fat)	–GD84–Weaning				↑ Adipocyte hypertrophy ↑ Macrophage infiltration	↑ Inflammatory cytokines	↓ Glucose tolerance
Chang et al. [92]	Mice	Normal fat diet (13% kcal fat)	–GD 42–GD 0	Normal diet Vs HFD at 13 weeks of age	Male and female	24 weeks	↔	↔	↔
		HFD (60% Kcal fat)		Normal diet			↔	↔	↓ Glucose tolerance (males only)
				HFD at 13 weeks of age			↑ SAT (male only)	↑ Increase CD11 macrophages (males only)	↓ Glucose tolerance (males only)
Murabayashi et al. [91]	Mice	HFD (60 kcal% fat) Vs chow diet	–GD28–Birth	NA	Not specified	Newborn	↑ Hypertrophic SAT	↑CD68, CCR2, and TNF-α mRNA levels ↓ GLUT-4 mRNA	
Fernandez-Twinn et al. [93]	Mice	HFHS diet Vs chow diet	–GD43–End of second lactation	Chow diet	F2 male offspring	8 weeks	NA	↓IR, AKT1, AKT2, p110β, p85α and IRS-1 protein levels ↑ miR-126	
Lemonnier et al. [96]	Mice	High-fat diet (40% fat) Vs normal diet	Confirmation of pregnancy–lactation	Same as respective maternal diet	Both	32 weeks	↑ Hyperplasia in perirenal AT (in males) ↑ Hyperplasia and hypertrophy in parametrial AT (in females) ↑ Hypertrophy in epididymal AT and SAT	NA	↑ Body weight
Snajder et al. [94]	Rats	Chow diet	–GD 42–Weaning	Chow diet Vs high-fat diet	Male	22 weeks old	↑ Subcutaneous and epididymal adipocyte size	NA	NA
		High-fat diet (rich in saturated fats)		Chow diet Vs high-fat diet			↑ Subcutaneous and epididymal adipocyte size ↑ Number of adipocytes	NA	NA
Lima et al. [90]	Rats	Normal fat diet (4% fat) vs high-fat diet (23% fat)	Conformation of pregnancy–Weaning	Lactation	Unspecified	22 days of age	↑ VAT	NA	↑ Weight gain ↑ Total cholesterol
Raipuria et al. [95]	Rats	High-fat diet (43% fat) Vs chow diet	–GD 42–GD 0	NA	Male and female	Day 19	↑ Adiposity	NA	↑ Body weight
		High-fat diet (43% fat) + exercise Vs chow diet + exercise					↑ Adiposity	NA	↑ Body weight

*Ad Lib* ad libitum feeding, *AT* adipose tissue, *BAT* brown adipose tissue, *GD* gestational day, *HFD* high-fat diet, *Kcal* kilocalories, *NA* not applicable, *SAT* subcutaneous adipose tissue, *VAT* visceral adipose tissue, *Vs* versus, *WAT* white adipose tissue.

Work in mice suggests that the duration of maternal high fat diet consumption has little effect on the magnitude of changes in offspring adiposity, as long as the diet was consumed within 9 weeks of mating [89]. Other work has shown that offspring of mothers consuming an obesogenic diet during gestation may be more prone to dietary-induced changes postnatally. For instance, rat offspring exposed to maternal obesity show greater morphological changes, including enhanced adipocyte hyperplasia in response to a postnatal high-fat diet [94]. Finally, some research suggests that programmed changes in offspring adipose biology may be permanent, as an exercise intervention in mothers fed a high-fat diet did not prevent the increased adiposity or trigger beneficial changes in insulin signaling component expression in their fetuses [95]. However, whether there are beneficial impacts of maternal exercise in ameliorating the impacts of maternal obesity/obesogenic diets on offspring later in life warrants investigation.

No studies have explored whether maternal obesity/obesogenic diets programme changes in offspring BAT. Similarly, whether maternal obesity/obesogenic diets programme offspring adipose biology and obesity in a sex-dependent manner have been under investigated. Of the studies that have, there are differences in adipose tissue morphology and leukocyte influx, as well as metabolic phenotype between exposed male and female offspring [92, 96]. However, the mechanisms underlying sexually dimorphic alterations in programmed outcomes remains unexplored.

### Hypoxia and offspring adiposity

Hypoxia can have many deleterious effects on a developing fetus and can be caused by a variety of situations, such as placental insufficiency, maternal anemia, smoking, and increased altitude [97, 98]. In humans, cigarette smoking in early pregnancy increased the likeliness of the infant being overweight at 3 years of age [99]. Experimentally, hypoxia can also be induced by placing animals in chambers where the availability of oxygen is reduced. The impact of hypoxia on the developing fetus depends on a variety of factors, including the severity of the hypoxic episode, duration, and stage of gestation, however these have not be studied in the context of programmed changes in offspring adipose biology and obesity risk. In the studies available (Table 4), exposure to hypoxia during gestation was linked to increased offspring adiposity in adulthood [100, 101]. This was related to an elevated expression of pro-inflammatory markers in the adipose tissue of hypoxia exposed rodent offspring compared to their normoxic counterparts [100]. Exposure to hypoxia during intrauterine development was also associated with molecular changes, including enhanced UCP1, DIO, and PPARγ expression in the adipose of sheep fetuses, suggesting the adipose may be programmed towards the beige phenotype [102]. However, further work is required to assess the impact of gestational hypoxia and influence of offspring sex.

**Table 4:** Maternal hypoxia and its effect on offspring adiposity.

Study	Species	Maternal manipulation	Duration	Offspring diet	Offspring sex	Offspring age	Adipose structure	Adipose molecular	Whole body changes
Badran et al. [142]	Mice	Intermittent hypoxia (21–12% hypoxia cycles 60 times a day) Vs control (room air)	GD1–GD 18	Chow	Male and female			↑ DNA methylation of CpG islands of adiponectin gene promoter in PVAT of males	↓ Body weights ↑ Plasma lipids ↑ Leptin ↑ Insulin resistance in male adult offspring
Khalyfa et al. [101]	Mice	Intermittent hypoxia (21–6% hyposia cycles 20 times a day) Vs control (room air)	GD13–GD18		Male and female	24 weeks	↑ Adiposity	↑ Macrophages in WAT differential methylation patterns including in PPARy	↑ Body weight
Vargas et al. [100]	Rats	Hypoxia (12% O2) Vs normoxia	GD15–GD19	Chow HF	Male	25 weeks	↑ Peritoneal fat ↑Peritoneal fat	↑ IL-1β, TNFα, and IL-6 mRNA	
Myers et al. [102]	Sheep	3820 m above sea level Vs 346 m above sea level	GD30–GD137/138	NA	Male and female	GD 140		↑ UCP1, HSD11β, PPARy, PGC1, DIO1, and DIO2	

*Ad Lib* ad libitum feeding, *GD* gestational day, *m* meters, *NA* not applicable, *Vs* versus.

### Endocrine disruptors and offspring adiposity

In recent years, there has been an increasing body of research into the effects of endocrine disruptors on human health and development. The focus of these studies is on synthetic chemicals known as persistent organic pollutants due to their resistance to environmental and biological degradation and accumulation in the environment and biological tissues [103]. These include; bisphenol A (BPA), which is found in clear plastics, and phalates (such as di-2-ethylexyl phalate, DEHP), which are found in products, such as shampoos, moisturizers, and liquid soaps. Both these chemicals can disrupt biological processes by acting as endocrine disruptors. There is accumulating evidence from human and animal studies that shows associations between exposure to organic pollutants and other widely diffused toxins to an increased risk of metabolic diseases, including obesity [104]. These synthetic chemicals are thought to promote obesity in children and adults by increasing adipogenesis [105, 106]. There is evidence that BPA can traverse the placenta and reach the fetus in humans and other species [107, 108]. In humans, maternal urinary BPA has been associated with increased hip to waist ratios in the offspring, an affect that was more severe in females [109].

Animal studies of BPA and phalate exposure in utero have consistently shown to increase offspring adiposity in later life (Table 5 and [106, 110–112]). In part, these changes are associated with enhanced adipocyte size, infiltration of inflammatory leukocytes, and elevated expression of lipogenic markers, like PPAR-γ and SREBP [113]. Developmental exposure to BPA has also been linked to enhanced levels of oxidative stress in the adipose of exposed offspring however, such an effect was not prevented by exposure to a perinatal Mediterranean diet that is known to offer protection against oxidative stress [114]. There may also be a greater number of adipocytes in the BAT of adult mouse offspring exposed to phalate during gestational and lactational development [111]. Likely due to their endocrine disrupting effects, BPA and phalates have been shown to exert sex-dependent impacts on offspring adiposity [106, 110]. However, due to the low number of studies that have specifically looked at sex-specific effects, firm conclusive results are yet to be made. Given the available data and their persistence in tissues, organic pollutants, like BPA and phalates possibly play a key role in the rapid epidemiological growth of obesity. However, more studies are required to examine this.

**Table 5:** Maternal exposure to endocrine disruptors and its effect on offspring adiposity.

Study	Species	Maternal manipulation	Duration	Offspring diet	Offspring sex	Offspring age	Adipose structure	Adipose molecular	Whole body changes
Lee et al. [111]	Mouse	30 mg/kg DEHP Vs control	–GD 2–Weaning	Chow	Male and female	8 weeks	↑ Adiposity ↑ Size of adipocytes in WAT ↑ Number of adipocytes in BAT	NA	
Hunt et al. [112]	Mouse	0.05 mg/kg/day DEHP Vs control	GD0–Weaning	HFHS		22 weeks	↑ Adipogenesis	NA	↑ Insulin sensitivity
Malaise et al. [113]	Mouse	50 μg/kg BW/day of BPA Vs control	GD 15–Weaning	Chow	Males	170 days old	↑ Infiltration of pro-inflammatory M1 macrophages in gonadal WAT	NA	↓ Insulin sensitivity ↑ Weight gain
Neier et al. [114]	Mouse	Mediterranean HFD Vs control (7% corn oil control chow)	–GD14–Weaning	7% corn oil control chow	Male and female	10 months	NA	↑ Oxidative stress in mWAT	
		Western HFD Vs control (7% corn oil control chow)					NA	↔	
		7% corn oil control chow +50 μg BPA/kg Vs control (7% corn oil control chow)					NA	↑↑ Oxidative stress in mWAT	
		Mediterranean HFD + 50 μg BPA/kg chow Vs Mediterranean HFD					NA	↑↑ Oxidative stress in mWAT	
		Western HFD + 50 μg BPA/kg chow Vs HFD					NA	↑↑ Oxidative stress in mWAT	
Somm et al. [110]	Rats	1 mg/L BPA in water Vs control	GD6–Weaning	NA	Males and females	21 days old	↑ Parametrial WAT weight ↑Adipocyte hypertrophy	↑ C/EBP-α, PPAR-γ SREBP-1C, LPL, FAS, and SCD-1	↑ Body weight (in females)
Desia et al. [106]	Rats	BPA (5 mg/l in water) Vs control	–GD14–Weaning	Chow	Male and female	1 week, 3 weeks, and 24 weeks	↑ Adiposity in males Hypertrophic adipocytes	At 1 week ↑ PPARγ (in males) At 3 weeks ↑C/EBPα, SREBP1, CD68, TNFα (in males)	↑ Body weight (in males)

*Ad Lib* ad libitum feeding, *BAT* brown adipose tissue, *BPA* bisphenol A, *BW* body weight, *DEHP* Di-2-ethylexyl phalate, *GD* gestational day, *HFD* high-fat diet, *HFH*S high fat high sugar diet, *NA* not applicable, *SAT* subcutaneous adipose tissue, *VAT* visceral adipose tissue, *Vs* versus, *WAT* white adipose tissue.

### Glucocorticoids and offspring adiposity

Glucocorticoids are a class of steroid hormones that are part of the hypothalamic pituitary adrenal axis and play an important role in fetal development, including the maturation of fetal organs prior to birth [115, 116]. However, excess exposure to glucocorticoids during gestation can slow growth and development of the fetus with negative effects on offspring health and developmental programming. Maternal stress, anxiety and depression during pregnancy have been shown to increase fetal exposure to glucocorticoids [117]. The use of synthetic glucocorticoids to advance lung maturation in pregnancies with threatened preterm birth also increases fetal exposure to glucocorticoids. In humans, maternal second trimester levels of corticotropin-releasing hormone, a hormone that stimulates the synthesis of glucocorticoids, are positively correlated with central adiposity in infants at 3 years of age [118]. In animal studies, excess glucocorticoid exposure in utero was consistently shown to programme the offspring for increased adiposity (Table 6 and [119–123]). This was coupled with alterations in adipocyte morphology, expression of lipogenic genes, insulin signaling proteins and inflammatory cytokine expression in the offspring WAT [124]. There were also cellular and molecular changes in the BAT of offspring over-exposed to glucocorticoids prenatally, namely decreased UCP1 expression, increased lipid droplet size, upregulated prolactin receptor and decreased mitochondria content, which would be expected to compromise tissue function [64, 125]. However, sex effects have not been explored and further work is required to identify the importance of length, timing and level of glucocorticoid over-exposure during gestation to the developmental programming of offspring adipose biology and obesity.

**Table 6:** Excess glucocorticoids during gestation and its effect on offspring adiposity.

Study	Species	Maternal manipulation	Duration	Offspring diet	Offspring sex	Offspring age	Adipose structure	Adipose molecular	Whole body changes
Chen et al. [125]	Mouse	Dexamethasone (0.1 mg/kg body weight) Vs control (PBS)	GD14–GD20	Chow diet	Male	4 Months	↓ BAT ↑ Lipids in gonadal and inguinal fat ↑ Sizes of lipid droplets in BAT ↓ Mitochondrial number in BAT	↓ UCP1 in BAT ↓ pIRS-1 (T612) and pAKT (S473) ↑DNA methylation in Pparγc1a promoter	↓ Insulin sensitivity
Sugden et al. [160]	Rat	100 or 200 mg/kg dexamethasone maternal body weight per day Vs control	GD15–GD21	Chow diet	Male and female	12 weeks and 1 year	NA	NA	Hyperleptinaemia
Zulkafli et al. [121]	Rats	0.5 μg/mL of dexamethasone Vs control	GD13–Term	Chow diet	Male		↑ Epididymal fat		↑ Adiposity
				HFD (45% fat)			↑ Epididymal fat		↑ Adiposity
				High fat high omega-3			↑ Epididymal fat		↑ Adiposity
Mark et al. [124]	Rats	Dexamethasone (0.75 μg/mL in drinking water) Vs control	GD13–Term	Cross fostered to chow diet mother	Male and female	6 months	↔	↑ Il6, Il1β, Tnfα, GR, and Pparα	↑ Serum fatty acid levels
				Cross fostered to mother with diet high in omega 3	Male and female	6 months	↓ Adipose size	↑ GR and Pparaα	
Wyrwoll et al. [161]	Rats	Dexamethasone (0.75 μg/mL in drinking water) Vs control	GD13–Term	Cross fostered to chow diet mother	Male and female	6 months	NA	↑ ACE in retroperineal fat	
				Cross fostered to mother with diet high in omega 3	Male and female	6 months	NA	↔	
Dahlgren et al. [122]	Rats	Dexamethasone (intramuscularly 100 mg/kg) VS control		Chow diet	Male and female	11 weeks	↑ Retroperitoneal fat depot mass ↑ Parametrial fat depot mass in females		↔ Body weight
Long et al. [119]	Sheep	Dexamethasone (4 injections of 2 mg intramuscularly, 12 h apart) Vs control (saline)	GD 103 onwards	Ad libitum	female	F2 at 13 months	↑ Adiposity	NA	↑ Feeding impaired insulin secretion
Blasio et al. [120]	Sheep	Dexamethasone (0.48 mg/h) Vs control saline (0.19 mL/h)	Intravenous infusion for 48 h from GD 26–GD 28	NA	Male	4 years	↑ Adiposity	NA	First-phase hyperinsulinemia
		Cortisol (5 mg/h) Vs Control saline (0.19 mL/h)					↑ Adiposity	NA	Second-phase hyperinsulinemia
Bispham et al. [64]	Sheep	16 mg dexamethasone Vs control	GD138	NA	Male and female	6 h post birth		↑ Abundance of the 54 and 48 kDa isoforms of PRLR in BAT	
Weiler et al. [123]	Pigs	Dexamethasone (administered in milk) Vs control	Day 5–Day 20 of suckling		Males	21 days old	↑ Fat mass		↓ Weight ↓ Bone mass

*Ad Lib* ad libitum feeding, *BAT* brown adipose tissue, *GD* gestational day, *HFD* high-fat diet, *NA* not applicable, *PBS* phosphate buffered saline, *SAT* subcutaneous adipose tissue, *VAT* visceral adipose tissue, *Vs* versus, *WAT*, white adipose tissue.

### Androgens and offspring adiposity

Androgens are steroid hormones that typically regulate the development and maintenance of male characteristics. Possibly the most well-known and typically investigated androgen in developmental studies is testosterone. As a lipid-soluble hormone, testosterone is thought to be able to cross the placenta and this notion is supported by data showing that fetal plasma testosterone concentration is positively correlated with maternal testosterone [126]. For obvious reasons, in utero androgen exposure is particularly harmful to female offspring. There have been many studies showing that excess testosterone in utero contributes to the development of polycystic ovary syndrome (PCOS), which is estimated to affect 10–20% of women in the UK [127]. Therefore, androgen exposure in utero is thought to be both a cause and a consequence of PCOS, impacting the transmission of the disease [128]. PCOS increases the risk of type 2 diabetes and obesity in women, thus in utero androgen exposure may both directly and indirectly have an impact on adiposity in later life [129, 130]. However, PCOS is not the only cause of excess androgen exposure in utero; female dizygotic twins may also be exposed due to excess androgens from their male twin during gestation. Moreover, maternal testosterone concentration has been shown to negatively correlate with birth weight and BMI in female offspring [131].

As shown in Table 7, work in experimental animals has shown that excess androgen exposure in utero or in early neonatal life programmes the female offspring to have changes in adipose tissue biology [132–135]. There is a change in the size of WAT adipocytes [132, 136] and an increase in chemokine and pro-inflammatory cytokine expression and dysregulated adiponectin expression, depending on the timing of the androgen exposure and WAT depot in female offspring [132, 137]. Androgen exposed female offspring also seem to have an additional increase in pro-inflammatory cytokines when fed an obesogenic diet postnatally [137]. Exposure of female offspring to testosterone neonatally leads to increased BAT which is dysfunctional [136], although whether prenatal exposure also affects BAT phenotype postnatally has yet to be explored. There is also a greater sensitivity of androgen exposed female offspring to increase their body weight with an obesogenic diet postnatally [137]. However, further work is required to identify the importance of timing, duration, and level of androgen over-exposure during gestation, versus in the neonatal period to the programming of offspring adiposity, in relation to the pathogenesis of metabolic disorders like obesity and PCOS.

**Table 7:** Excess androgens during gestation and its effect on offspring adiposity.

Study	Species	Maternal manipulation	Duration	Offspring diet	Offspring sex	Offspring age	Adipose structure	Adipose molecular	Whole body changes
Nohara et al. [136]	Mice	Testosterone (100 μg) Vs control	Neonatal days 1 and 2	Chow diet	Female	6 weeks	↑ Dysfunctional WAT ↑ Number of enlarged insulin-resistant adipocytes ↑ Dysfunctional BAT ↑ BAT mass ↓ Energy expenditure.		Leptin resistance hypoadiponectinemia.
Gulan et al. [137]	Rats	Testosterone (0.5 mg/g/day) Vs control	GD15–Term	Chow diet	Female	8 weeks		↑ Pro-inflammatory cytokines (IL-1β, IL-18, and MCP-1)	↑ Body weight ↑ Serum levels of testosterone, insulin, and leptin ↓ Adiponectin levels
				HFHS diet (compared to chow control)				↑ Pro-inflammatory cytokines (IL-1β, IL-18, IL-6, TNF-α and MCP-1)	↑ Body weight
Nilsson et al. [134]	Rats	Testosterone (1 mg) Vs control	1 injection 3 h after birth	Chow diet	Female	10 weeks	↓ Parametrial, retroperitoneal, and inguinal adipose tissue mass ↑ Mesenteric adipose tissue mass	NA	↑ Body weight
Xu et al. [133]	Rhesus Monkeys	Testosterone (1 mg) Vs control	40 consecutive days beginning on GD40–GD44		Female	9 weeks	NA	163 differentially methylated CPG islands in VAT Altered pathways included TGF-β, Wnt/ β-catenin, and antiproliferative signaling	
Puttabyatappa et al. [132]	Sheep	Testosterone (100 mg) Vs control	GD30–GD90 twice weekly	Standard diet	Female	21 months	↓Adipocyte size	↓ ADIPOQ mRNA in VAT ↑ ADIPOQ mRNA in SAT ↑ TNF-a, CCL2 mRNA in VAT and SAT ↑ IL1B mRNA in VAT	
Lu et al. [135]	Sheep	Testosterone (100 mg) Vs control	GD30–GD90 twice weekly	Standard diet	Female	2 years	NA	↑ p-mTOR	

*Ad Lib* ad libitum feeding, *BAT* brown adipose tissue, *GD* gestational day, *HFD* high-fat diet, *NA* not applicable, *SAT* subcutaneous adipose tissue, *VAT* visceral adipose tissue, *Vs* versus, *WAT* white adipose tissue.

### Programming mechanisms of adipose tissue

The programming mechanisms behind changes in adipose remain elusive. The studies which do explore this broadly involve evaluating epigenetic changes in the adipose tissue and studying the adipogenic potential of mesenchymal stem cells from exposed offspring. However, as WAT develops postnatally in some species like rats and mice, changes induced by maternal manipulations may also be the result of alterations in other systems, particularly the hypothalamic pituitary adrenal hormonal axis in the offspring during gestation [138]. Indeed, as described above, there are links between glucocorticoid over-exposure in utero and the programing of obesity in the offspring.

#### Epigenetic mechanisms

Suboptimal maternal environments, particularly those which occur during critical developmental windows may permanently alter gene expression in offspring tissues via epigenetic mechanisms. Such mechanisms include changes in DNA methylation, histone modifications, and the expression of noncoding RNAs. Certainly, a number of studies exploring fetal programming of adipose tissue have suggested that each of these epigenetic processes may contribute.

In the WAT of males from obese rats, there are alterations in DNA methylation of CpG sites proximal to C/EBP-β and Zfp423 genes, which encode key transcriptional factors initiating adipogenic commitment and is consistent with the increased expression of key adipogenic regulators, PPAR-γ, C/EBP-α, and C/EBP-β [139]. In another study, exposure to maternal obesity was also associated with lower Zfp423 promoter methylation levels and increased Zfp423 gene expression in offspring adipose tissue in alliance with enhanced adiposity [140]. In addition, a maternal low protein diet throughout gestation and lactation lead to alterations in the methylation of CpG sites proximal to the leptin gene in the adult offspring [141] and likely contributed to the abnormalities in adipose tissue biology seen in other work on the model. Some studies have also shown that changes in offspring adiposity with gestational hypoxia are coupled with changes in DNA methylation patterns in the adipose tissue [101, 142]. There was also increased DNA methylation of the Ppargc1a promoter in neonatal BAT and brown adipocyte progenitor cells, which was linked with attenuated BAT development in response to glucocorticoid over-exposure during gestation [125]. Finally, the methylation of multiple genes was aberrant in the visceral WAT of infant and adult offspring exposed to excess androgens, which likely contributed to the development of obesity and PCOS in the experimental model [133].

Maternal obesity and high-fat diets have also been associated with alterations in histone modifications in the adipose tissue of adult offspring. Of note, maternal obesity was associated with increased H3K4me1/H3K27ac histone modifications in enhancer sites upstream of the leptin gene, which correlated with enhanced leptin expression in the WAT of male offspring [143]. Moreover, a maternal high-fat diet during pregnancy lead to alterations in histone modifications at the promoter regions of the adiponectin and leptin gene in the WAT of offspring [144].

Noncoding RNAs have also been shown to play a role in the fetal programming of adipose tissue. For example, maternal obesity was associated with upregulated expression of miR-126, which negatively regulates insulin receptor susbtrate-1 (IRS1) and this was correlated to reduced IRS1 abundance and insulin sensitivity of the WAT in exposed offspring [93]. Furthermore, maternal low protein diets during gestation were associated with an increase in miRNA-483-3p levels, which is known to reduce adipose adipogenesis [145].

#### Mesenchymal studies

To inform on the programming of obesity, mesenchymal stem cells have been harvested from the umbilical cord of offspring exposed to different in utero/maternal environments. Previous work has shown there is a positive correlation between the preference of mesenchymal stem cell fate decisions towards the adipocyte lineage and infant adiposity [88, 146]. In addition, mesenchymal stem cells from infants of obese mothers showed a decrease in the abundance of β-catenin, which would favor adipocyte rather than myocyte differentiation [88]. Consistent with this, the differentiation of mesenchymal stem cells to adipocytes in vitro was increased and adiposity greater in 5-month-old infants from obese mothers [88]. Higher adiposity in infants of obese mothers was also linked to increased lipid species content and lipid transport gene expression in differentiated adipocytes and elevated oxidative stress, lower amino acid concentrations and expression of growth-promoting genes in their differentiated neonatal myocytes [146].

### Summary and conclusions

Alterations to the in utero environment as a result of suboptimal maternal conditions are related to programmed changes in the development, structure, and function of adipose tissue in the offspring. A variety of nutritional manipulations and differing metabolic environments such as low protein, caloric/nutrient restriction, obesity/obesogenic diets have all been shown to impact offspring adipose structure and function. Non-nutritional changes, such as hypoxia and exposure to hormones and pollutants/synthetic chemicals have also been shown to affect adipose tissue in the offspring. The outcomes of these manipulations differ and are dependent on the manipulation itself, that is, its timing, duration, and severity. Across a variety of mammalian species, the impacts of certain manipulations, such as low protein diet remains relatively consistent increasing the reliability of this data. However, broadly speaking, while the majority of investigations available studied programmed changes in whole body metabolism, the adipose tissue was examined in a very limited capacity. The majority of studies also analyse only one fat depot and as discussed, these can vary greatly and are reflective of the organ they are associated with. In addition, certain adipose pads, particularly the subcutaneous adipose vary with offspring sex. The focus of investigations also tend to be on WAT so the effect of maternal manipulations on the programming of BAT development and function remains relatively unknown.

The impact of sex on the fetal programming of adipose tissue has been relatively under-studied. The majority of the studies only investigated male offspring and of the studies that do include female offspring, very few compare sexes. The studies which do compare sexes frequently find disparities in offspring outcomes. Given the multiple differences in adipose tissue between males and females previously discussed, this is unsurprising. However, the mechanisms underlying these disparities in outcome from the same in utero environment remain uninvestigated. There have been speculations that some sex differences may be partly due to sex-specific epigenetic modifications operating at the level of the placenta during development. The placenta is the functional interface between mother and fetus and the main determinant of maternal nutrient, oxygen, and hormonal supply to the fetus during gestation [9, 147]. Of note, Gallou-Kabani et al. showed that the global methylation pattern of the placenta was different between male and females, even though they were exposed to the same in utero environment [148]. However, whether a placenta-specific manipulation is sufficient to programme alterations in offspring adipose biology and obesity and do so, in a manner influenced by fetal sex, are yet to be studied. Indeed, previous work has shown that several of the maternal environmental manipulations surveyed here affect placental formation and function in association with changes in fetal growth and offspring phenotype [9, 147, 149–152]. The placenta likely plays a major role in mediating the programmed alterations in offspring adipose development and function, and hence, susceptibility to develop obesity.

While many studies examine if maternal environmental challenges are linked to changes in adiposity or adipocyte biology in the offspring, the mechanisms underlying these programming impacts remains largely unknown. Generally, the molecular analyses performed are targeted and focus on either adipose inflammation or insulin signaling pathways, with few studies undertaking wider capturing analyses, such as RNA sequencing, which would provide a greater understanding of underlying programming effects. In addition, mechanisms by which obesity may be programmed across generations is largely unexplored. Where there have been studies into transgenerational inheritance, some studies have found robust changes, suggesting a change in the offspring heritable epigenome. For example, in mice, female progeny of obese dams exposed to high-fat diet displayed obesity and high levels of WAT inflammation in association with hypomethylation at certain inflammatory genes for three generations [153]. Furthermore, investigations of the Dutch famine cohort found indications of transgenerational transmission of obesity in humans [154]. Thus, perhaps the most significant avenue for the future of this field, is designing studies to further elucidate the cellular and epigenetic mechanisms underlying fetal programming and transgenerational effects. This is important as the adipose tissue plays a fundamental role in whole body homeostasis and metabolism and the potential transgenerational inheritance of obesity maybe one of the factors contributing to its increase in many populations worldwide.

Given the current epidemic levels of metabolic diseases across the globe, the central focus of research in this area should be on understanding the mechanisms of fetal programming in a way to create an optimal in utero environment to prevent and alleviate disease transmission. This requires a better understanding of the impact of diet and hormone exposures on the developing adipose tissue in humans, as well as a greater knowledge of the influence these may have on critical events, such as mesenchymal stem cell fate decisions. It also requires a better understanding of the plasticity of adipose tissue across the life course, as this will help inform on what interventions may be beneficial in combatting the effects of a poor in utero environment on the developmental programming of offspring obesity risk. There have already been interesting studies in this area, including data showing that maternal dietary supplementation with methyl donors during gestation or lactation partially prevented the development of an obese phenotype in the offspring from high sucrose diet-fed mothers [155]. This “deprogramming” of offspring phenotype via such an approach may prove to be a promising strategy to overcome the transgenerational transmission of obesity and help curb the current obesity epidemic and the high costs to health services worldwide.
