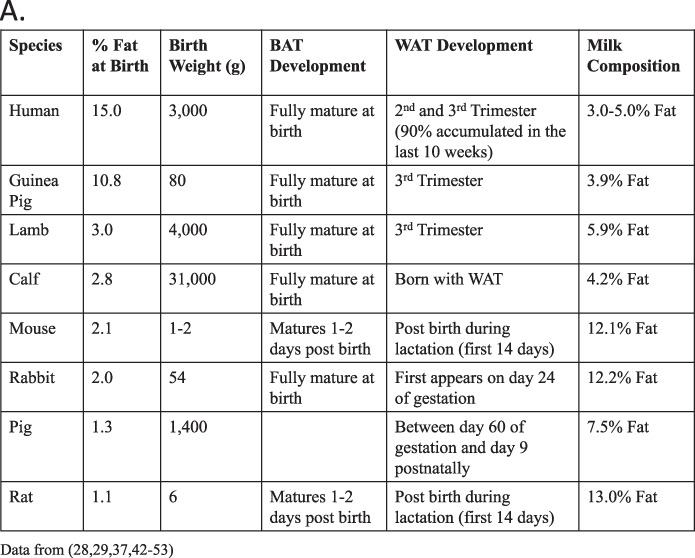# Correction to: Developmental programming of offspring adipose tissue biology and obesity risk

**DOI:** 10.1038/s41366-021-00828-z

**Published:** 2021-05-10

**Authors:** Amanda Rodgers, Amanda N. Sferruzzi-Perri

**Affiliations:** Centre for Trophoblast Research, Department of Physiology, Development and Neuroscience, Downing Street, University of Cambridge, Cambridge, UK

**Keywords:** Obesity, Fat metabolism

Correction to: *International Journal of Obesity*

10.1038/s41366-021-00790-w

The original version of this article unfortunately contained a mistake. There were typographical errors in Fig. 2A (the birthweight of the mouse should be 1–2 g and for the pig it should be 1400 g). The correct values are given in the figure below. The authors apologize for the error. The original article has been corrected.